# Burkholderia cepacia complex bacteremia: an outbreak investigation with epidemiological link to contaminated disinfectant

**DOI:** 10.1017/ash.2025.10254

**Published:** 2025-12-23

**Authors:** Rozina Roshan, Seema Irfan, Rida Tafveez, Nazleen Virani, Mehreen Shahid, Syed Faisal Mahmood

**Affiliations:** 1 Department of Infection Prevention and Hospital Epidemiology, Aga Khan University, Karachi, Pakistan; 2 Department of Pathology and Laboratory Medicine, Aga Khan University, Karachi, Pakistan; 3 Department of Medicine, Aga Khan University, Karachi, Pakistan

## Abstract

**Objective:**

To describe an outbreak of Burkholderia cepacia complex at a tertiary care hospital in Karachi, Pakistan, highlighting contributing factors, potential sources, and system-level gaps identified during the investigation.

**Design:**

Outbreak investigation.

**Setting:**

A 655-bed tertiary care teaching hospital in Karachi, Pakistan.

**Participants:**

All individuals who had positive blood cultures by non-lactose fermenting, oxidase-positive, Gram-negative rods that could not be further characterized.

**Methods:**

On September 26, 2020, the Department of Infection Prevention and Hospital Epidemiology (DIPHE) was notified of multiple positive blood cultures. An outbreak investigation was initiated, including chart reviews, laboratory analysis, environmental sampling, assessing central line insertion practices, and evaluating the manufacturing site. Clinical Laboratory Standards Institute (CLSI) guidelines were used for microbiological identification and susceptibility testing.

**Results:**

Thirty-five patients with positive cultures were identified between September 15 and October 22, 2020. While environmental sampling did not yield growth, significant breaches at the suppliers‘ facility were identified in chlorhexidine gluconate (CHG) storage and quality control. Although cultures of CHG were negative, the product’s withdrawal led to a marked decline in new cases. Moreover, while resources were unavailable for genomic testing, antimicrobial susceptibility patterns were similar in all the case strains, suggesting a common source.

**Conclusion:**

This outbreak highlights the role of contaminated disinfectants in healthcare-associated infections. It also revealed systemic gaps in disinfectant quality control, storage facilities, and diagnostic capacity, delaying outbreak recognition and response. It is essential to strengthen regulatory oversight, implement standardized testing protocols, and enhance microbiological diagnostic infrastructure to lower the risk of similar outbreaks.

## Introduction

The Burkholderia cepacian complex (BCC) is a group of Gram-negative bacteria initially thought to be a single species, but now compromises almost 24 opportunistic pathogenic species.^1^ It is widespread in the environment and demonstrates a high capacity for rapid mutation and adaptation posing significant threat to patients with cystic fibrosis (CF) and chronic granulomatous disease (CGD)^2^ due to intrinsic resistance to antimicrobial agents ^3^ and oxygen limitation. As opportunistic pathogens, infections vary from asymptomatic to more severe conditions, such as necrotizing pneumonia, or bacteremia in hospitalized patients. The bacterial genome consists of two to four replicons and several insertion sequences, providing genomic flexibility and rapid adaptation, making eradication difficult^3^. Moreover, due to resistance to antiseptics and disinfectants, BCC has the propensity to cause outbreaks^4^.It has been implicated in several outbreaks involving the bloodstream, respiratory tract, and urinary tract in intensive care units^5^ through contaminated environment, devices, and solutions, such as chlorhexidine (CHG), inhaled solutions, mouthwash, moisturizing creams, and ultrasound gels.^4^ On September 26, 2020, an outbreak of BCC bacteremia was observed in 35 patients at Aga Khan University Hospital (AKUH), Karachi, Pakistan. In this report, we describe this outbreak and its investigation.

## Materials and methods

### Setting

The Aga Khan University Hospital (AKUH) is a 655-bedded Private tertiary care teaching hospital in Karachi, Pakistan. It serves patients with various acute and chronic conditions including oncology patients. It has 36 and 16 oncology beds capacity for adults and children respectively, with 54 beds in daycare oncology service. On September 26, the clinical microbiology laboratory notified Department of Infection Prevention and Hospital Epidemiology (DIPHE), AKUH that several blood cultures were showing growth of an identical Gram-negative rod on colony morphology, biochemical characteristics and antibiotic susceptibility pattern.

### Epidemiological investigation

Based on above information, provided by clinical laboratory, DIPHE constructed a provisional BCC bacteremia outbreak case definition “Isolation of *Burkholderia cepacian* complex (BCC) with good zone of inhibition for trimethoprim-sulfamethoxazole and levofloxacin, no zone of inhibition for meropenem, and narrow zone of inhibition for ceftazidime, from blood culture of AKUH admitted/ enrolled patient.” In response to unusual clustering of cases an outbreak investigation was launched on September 30, 2020.

**Figure 1.**
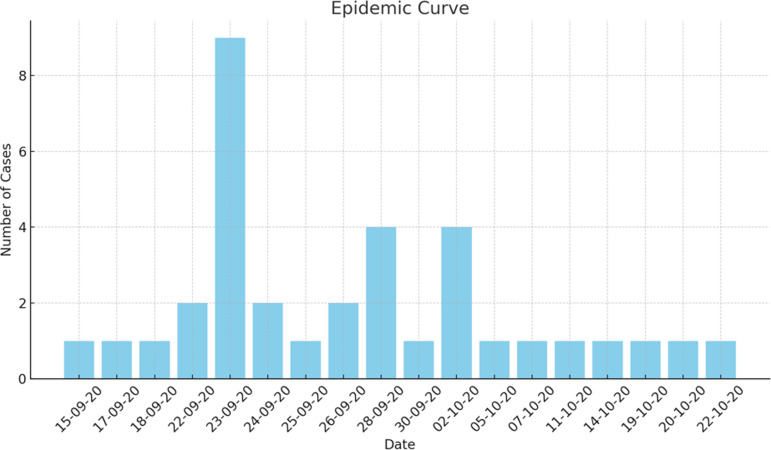
Epidemic curve of Burkholderia Cepacia complex outbreak.

Based on the available information, an interim case definition was formulated as positive blood cultures by non-lactose fermenting, oxidase-positive, Gram-negative rods that could not be further characterized. Subsequently, we identified 35 patients in total, including 22 cases that had occurred before the identification of this outbreak. Figure 1 shows the temporal distribution of Burkholderia cepacia complex cases during the outbreak. The case files of all the suspected patients were reviewed. Among 35 patients, data of 2 patients was missing, hence 33 patients were evaluated for their demographic and other characteristics as shown in Table 1. Variables associated with previous outbreaks caused by BCC were identified, such as the use of 70% isopropyl alcohol swabs, 2% w/v CHG with 70% isopropyl alcohol (CHG with alcohol), 70% alcohol hand rub, alcohol-free mouthwash, ultrasound gel, albuterol nebulization solution, distilled water used in nebulization, flushing orogastric tubes and humidification of oxygen were added in line listing. The DIPHE performed a field investigation. Based on the clinical review, several potential sources of contamination were evaluated randomly by doing culture (Table 2).

**Table 1.** Demographic and other characteristics of 33 patients with blood culture proven BCC bacteremia

Variable	Frequency *N* (%)
Adults	19 (57.5%)
Children	14 (42.4%)
**Admission due to Hematological malignancies**	**13**
B-cell/T-cell	5
High risk C-ALL	2
Leukemia	2
AML	2
Burkitt lymphoma	1
**Admission due to other malignancies**	**4**
DM spine malignancy	1
Breast cancer	1
Neuroblastoma (high risk)	1
Osteosarcoma	1
**Admission due to other diagnosis** (Pneumonia/meningitis/liver abscess/skull fracture, burn)	**6**
**Other comorbidities**	**13**
HTN	4
Diabetes mellitus	5
IHD/ CVA	3
CKD	1
Thalassemia major	1
Others (Potts disease, psoriasis, term/AGA/TTN)	4
**Total blood culture positive samples for BCC**	**42**
1 Sample	29
2 Samples	5
3 Samples	1
**Patient distribution in hospital**	**33**
Special care unit (SCU)	06
Intensive care unit (ICU)	08
General ward	12
Emergency department	04
Bone marrow transplant	02
Outpatient department	01

Note. Data of 2 patients was missing.

**Table 2.** List of samples sent for environmental culture

	Environmental culture	Date of sample received	Number of samples	Culture result
1	2% chlorhexidine with 70% alcohol solution (CHG with alcohol)	October 01, 2020	17 bottles of 4 lots	No growth
2	Sterile distill water	October 01, 2020	3 bottles	No growth
3	Sterile water	October 01, 2020	1 bottle	
4	Sterile normal saline	October 05, 2020	10 vials	No growth
5	Blood culture bottle	October 01, 2020	10 bottles LOT# 1055308 10 bottles LOT# 4055116	No growth
6	Alcohol pad	October 05, 2020	6 pads	No growth
7	Pair of gloves	October 06	2 pairs	No growth
8	Cannula stopper	October 07	05 stopper	No growth
9	Used sterile saline	October 05, 2020	05 vials from ED/D0/C2	No growth
10	Liquid soap	October 05, 2020	3 RTC from D2 /BMT 01 from ER	No growth
11	Used Purell (70% isopropyl alcohol)	October 05, 2020	1 bag	No growth
12	Used Surgillium solution (70% isopropyl alcohol)	October 05, 2020	1 bottle	No growth
13	Used ultrasound sound gel	October 01, 2020	1 sample radiology PICC line	No growth
14	Used multidose heparin vials	October 01, 2020	2 vials	No growth

### Microbiological investigation

The increase isolation frequency of a specific GNR from blood culture was communicated after ruling out pseudo-outbreak in two respects; firstly, bottle lot numbers were assessed for commonality and sterility. Two batches (0001055308 and 0004055116) of BacT/ALERT (Biomerieux) blood culture bottle lots were in use and have been provided with a certificate of Analysis by the Biomerieux. Additionally, on receiving at AKUH clinical laboratory, both lots have been verified for sterility (College of American Pathologist (CAP) standard). The bottles were then distributed to various hospital units, hospital laboratories as well as various sample collection units (n = 300) all over the country. In the current outbreak suspicion, lot sterility was further examined; 10 randomly selected uninoculated bottles from each lot were incubated in BacT/ALERT machine for 5 days. Secondly, epidemiological links were evaluated for any commonality regarding blood sampling unit/area, date, rate and pattern of positivity. This exercise ruled out blood cultures received from outside AKUH patients and indicated that outbreak cases belonged specifically to AKUH admitted patients.

All GNR isolates grew aerobically at 35°C on sheep blood agar (SBA) and chocolate agar plates with inhibited growth on MacConkey’s agar medium. All GNR isolates were oxidase test positive but showed inert reaction on conventional biochemical tests. The analytic profile index (API 20 NE (BioMérieux) identified 8 isolates as BCC while remaining were not identified as BCC. API NE of all strains not identified as BCC showed a profile number of 0045555 and interpreted as *Pseudomonas fluorescens* (63%) and *Ralstonia* (34%) with low discrimination. To identify further COWAN AND STEEL’S manual for the identification of medical bacteria (3^rd^edition) and Koneman’s color atlas and textbook diagnostic microbiology (Sixth edition) were referred, and these isolates were reported as BCC.

The susceptibility testing of BCC isolates was performed according to susceptibility testing guideline of Clinical Laboratory Standards Institute (CLSI M100, 30^th^ Edition, 2020).

17 bottles of unused CHG with alcohol belonging to the 4 currently in use batches on AKUH admitted patients were cultured. For culture, disinfectant solution was diluted and inoculated into aerobic BacT/Alert culture bottle and incubated for 10 days in BacT/Alert automated system. Given the inherent difficulties in culturing disinfectants and the ongoing suspicion of CHG with alcohol as a source of BCC outbreak, a multidisciplinary team comprising ID and microbiology experts and representatives from the hospital purchase and pharmacy departments visited the storage facility, and a detailed assessment was performed.

## Results

### Clinical features

Between September 15 to October 22, 2020, a total of 42 BCC isolates grew from 42 blood samples of these 35 patients. Data of 33 patients revealed that one-third of patients had malignancies. However, 15% of patients were admitted because of pneumonia, viral meningitis, skull fracture, electric burn, and liver abscess. Additional comorbidities included diabetes mellitus (DM), hypertension (HTN), and ischemic heart disease (IHD). The outbreak affected six units of the hospital (Table 1). 78.7% of patients were symptomatic while 21.2% were asymptomatic. Of the 35 patients, levofloxacin was the most frequently (60%) administered antibiotic followed by meropenem (20%), whereas co-trimoxazole was administered to only 2.85% of the patients. Notably, ceftazidime was not administered. Of these, two patients in the cohort succumbed to their underlying conditions. One patient, a known case of high-grade T-cell lymphoma, was initiated on chemotherapy before death. The second patient developed hypoxic respiratory failure, likely resulting from COVID-19 infection accompanied by bicytopenia, and death was attributed to disease progression. The patient had no preexisting comorbidities.

### Epidemiological investigation

No common environmental source/ procedure/ personnel were identified. However, the presence of a central line was found common in all cases (100%). Regarding line removal status of 35 patients, 16 patients (45.71%) had their lines removed within the last 10 days, whereas 19 patients (52.78%) retained their lines beyond this period. Given the strong association with central lines, we evaluated central line insertion practices in multiple units. A few breaches were identified; however, these differed across units, and no consistent pattern was observed. As 2% CHG with 70% isopropyl alcohol antiseptic solution (CHG with alcohol) was universally used for skin disinfection during central line insertion and maintenance, it was identified as a potential source of BCC bacteremia. The current batch of 2% CHG with alcohol was removed from each unit on October 28, 2020. However, its culture did not exhibit any growth.

During the storage facility audit performed on October 9, 2020, several deviations from recommended practices were identified. There were no standard operating procedures (SOPs) in place for quality improvement, nor were there any routine processes for conducting quality inspections to identify gaps and implement corrective/preventive actions. Vendor and lab quality control certification details were not available, and the facility lacked temperature monitoring. Containers and drums within the storage area were unlabeled, leading to organizational and traceability issues. Additionally, the overall sanitation and hygiene conditions are severely inadequate, posing significant risks for the storage of medical products. The bottles used for filling were unsealed and rinsed with tap water before filling, raising concerns regarding contamination. Furthermore, the facility was located near a garbage disposal area, increasing the risk of environmental contamination.

### Microbiological investigation

No growth was identified in environmental samples (Table 2). Sterility culture of uninoculated blood culture bottles from currently in use lots also showed no growth. All Outbreak BCC isolates exhibited uniform susceptibility patterns by Disc diffusion method; showed a substantial zone with trimethoprim-sulfamethoxazole, ciprofloxacin, intermediately sensitive or resistant to ceftazidime and no zone with imipenem, meropenem, aminoglycosides, and polymyxin B. Vitek susceptibility confirms these findings with an additional susceptibility to levofloxacin (Table 3). This susceptibility pattern was changed from BCC strain isolated routinely from clinical samples at AKUH laboratory, that show sensitivity to ceftazidime, meropenem, ciprofloxacin, levofloxacin and trimethoprim-sulfamethoxazole and resistant to polymyxin B, aminoglycosides, and imipenem.

**Table 3.** Susceptibility pattern of *Burkholderia cepacian complex* (BCC) outbreak isolates (*N* = 42)

	Antimicrobial	Sensitive *N* (%)	Intermediate *N* (%)	Resistant *N* (%)
1.	Ceftazidime	–	38 (90.5)	4 (9.5)
2.	Levofloxacin	41 (100)	–	–
3.	Trimethoprim-sulfamethoxazole	42 (100)	–	–
4.	Meropenem	–	–	42 (100)
5.	Minocycline	3 (100)	–	–

### Interventions and measures taken

Once BCC outbreak was recognized, infection preventionists (Ips) increased their ward round frequencies to stress the standard precaution particularly hand hygiene. Moreover, central line insertion and maintenance bundles were re-emphasized.

Based on the audit results, 2% CHG with alcohol was immediately removed from the hospital. The process took a few days, as the units had previously issued bottles that were not accounted for. However, the number of cases dropped dramatically over the next four weeks, and the last case was found on October 22. This slow decline was also attributed to previous exposure to 2% CHG with alcohol prior to withdrawal.

## Discussion

We describe an outbreak of BCC bacteremia caused by contaminated 2% CHG with alcohol solution. All affected patients had central lines, with most being asymptomatic, or mild illness. No deaths were attributable to BCC. Epidemiologically, the outbreak was associated with 2% CHG with alcohol, as withdrawal of the product lead to abrupt decline in cases, although definitive causality could not be established due to negative cultures and the absence of molecular typing.

This outbreak highlighted significant challenges faced by healthcare facilities in low- and middle-income countries (LMICs), where epidemiological evidence rather than positive cultures, pointed toward a contaminated disinfectant source. BCC is an opportunistic pathogen with clinical manifestations ranging from no symptoms to severe respiratory infections and septicemia.^6^ Infections and asymptomatic colonization with BCC are common in patients with cystic fibrosis, postoperative wound infections, pneumonia, urinary tract infections, and septicemia.^7^ Consistent with previous reports, our cohort had a predominance of oncology patients and relatively mild clinical outcomes, aligning with the patterns observed in similar immunocompromised patients. Hence, correlating with literature that many BCC strains are of low virulence and frequently cause transient or asymptomatic bacteremia in vulnerable populations.

The association with the CHG product is supported by several observations; universal use across all units, absence of alternative common exposures, negative cultures from other solutions, and abrupt cessation in cases after the product withdrawal. Similar outbreaks associated with contaminated disinfectants, including CHG have been reported.^8,9^ One of the issues related to poor disinfectant quality is the suboptimal concentration of disinfectants in the locally available disinfectant/antiseptic preparations. This could lead to the ineffective killing of microorganisms, resulting in their survival and propagation. This was highlighted by Mal et al., who demonstrated that locally purchased CHG digluconate preparations had a higher minimum inhibitory concentration (MIC) against clinical Gram-negative and Gram-positive clinical isolates when compared to control preparation of CHG (Sigma, St. Louis, MO, USA).^10^ This study raised concerns regarding suboptimal disinfectant concentrations in local preparations. In addition to compromised product quality, improper use of disinfectants in healthcare facilities in LMICs further diminishes their effectiveness. Additionally, incorrect disinfectant dilutions resulting in prolonged use, and a lack of formal IPC training can further compromise disinfectant efficacy and increase MICs for various pathogens.

This outbreak highlights the importance for vigilance for atypical, non-fermenting bacteria in patients with central lines, even when clinical symptoms are mild.

Antimicrobial management was further complicated by resistance patterns, emphasizing the need for strong stewardship and tailored therapy. In our cohort, the observed susceptibility to trimethoprim-sulfamethoxazole and fluoroquinolones is consistent with previously reported epidemiology of BCC,^11^ and supports their role as empiric options when BCC bacteremia is suspected. Moreover, from an IPC perspective, strict adherence to central line maintenance, early recognition of atypical pathogen patterns, and timely escalation to outbreak investigation remain essential.

One of the key findings of this outbreak investigation was the identification of potential deficits in disinfectant manufacturing and quality control. Contaminated disinfectants act as reservoirs for healthcare-associated infection outbreaks in LMICs.^12^ Poor quality disinfectants are a recognized issue in LMICs,^13^ where regulatory oversight and manufacturing standards are often lax. Despite its widespread use, the quality of 2% CHG with alcohol in LMICs is often not rigorously regulated, as evidenced by the outbreak. The implicated product was stored under inadequate conditions at the packaging facility, with poor hygiene, lack of standardized procedures, and improper temperature control, considering temperatures exceeding 40°C in Karachi. These gaps highlight the vulnerability of local manufactures and hospital procurement systems. Strengthening procurement processes, implementing routine quality checks, and establishing daily temperature monitoring are essential steps to prevent recurrence.

The effectiveness of such measures depends on the capacity to rapidly detect and respond to failures in infection-prevention systems. Early detection of hospital-associated outbreaks relies on real-time hospital surveillance systems which facilitate prompt identification and containment of infectious diseases.^7^ Such systems are particularly important for monitoring infection rates in hospitals treating immunocompromised patients. However, establishing a real-time surveillance system in LMICs remains challenging. First, there is a need for trained healthcare and IPC personnel to monitor, identify, and respond promptly to outbreaks. Moreover, an integrated healthcare system with adequate and automated data flow and appropriate diagnostic facilities is required^14^ along with reference laboratories to perform disinfectant testing. Even in the presence of this, the diagnostic capabilities of LMICs are limited, which further delays the identification of an outbreak.

Integral to effective surveillance is the capacity for accurate and timely pathogen identification, which remains a major limitation of many LMIC laboratories. We faced a significant challenge during this outbreak investigation in the identification of BCC owing to the use of the analytic profile index (API 20NE), which is an older identification method with a limited identification profile. In LMICs, limitations in clinical laboratories adversely affect surveillance of infections and antimicrobial resistance. The capacity to identify pathogens at the species level using automated systems such as the analytic profile index, Vitek 2 ®, or BD Phoenix ® is limited,^15^ resulting in delayed or inaccurate diagnosis and ineffective treatment.^16^ Outbreak recognition was delayed by the initial misidentification of BCC as a pseudomonas species, highlighting the critical role of accurate diagnostics such as MALDI-TOF in effective outbreak management. Limited laboratory capacity for molecular strain typing using sequencing techniques further exacerbated this issue. Accurate identification of BCC requires advanced molecular methods. Therefore, it is essential to address this gap by improving laboratory training, investing in microbiological infrastructure and integrating molecular diagnostic tools to identify multi-drug resistance outbreak, thereby improving outbreak response time.

The study has several limitations. Strong circumstantial evidence implicated the disinfectant as the source of the outbreak. However, we were unable to confirm that CHG was the source because of the antimicrobial properties of the solution. Our capacity was limited by the inability to perform molecular typing. Furthermore, some clinical data was incomplete and environmental sampling was only done after the outbreak recognition, that may have missed earlier contamination. Despite certain limitations, the investigation demonstrates significant strengths, including a multidisciplinary approach, integration of microbiological, clinical and supply-chain findings, and rapid outbreak containment.

This outbreak highlights the critical vulnerabilities within healthcare systems in LMICs. Specifically, inadequate regulatory oversight, poor disinfectant quality, inappropriate use, limited diagnostic capacity, and insufficient surveillance infrastructure collectively contributed to the emergence and delayed outbreak recognition. To enhance patient safety and prevent similar incidents, it is essential to strengthen procurement processes, establish regulatory frameworks, invest in microbiological and diagnostic infrastructure, and implement robust infection prevention and surveillance systems.
